# Imagined Steps: Mental Simulation of Coordinated Rhythmic Movements Effects on Pro-sociality

**DOI:** 10.3389/fpsyg.2017.01798

**Published:** 2017-10-13

**Authors:** Liam Cross, Gray Atherton, Andrew D. Wilson, Sabrina Golonka

**Affiliations:** ^1^Psychology, School of Science, University of Buckingham, Buckingham, United Kingdom; ^2^Department of Psychology, School of Science and Technology, Sunway University, Selangor, Malaysia; ^3^Department of Psychology, Health and Learning Sciences, University of Houston, Houston, TX, United States; ^4^Psychology, School of Social Sciences, Leeds Beckett University, Leeds, United Kingdom

**Keywords:** coordinated rhythmic movement, interpersonal entrainment, interpersonal synchrony, interpersonal coordination, rhythmic entrainment, mental simulation, joint action, imagined contact

## Abstract

Rhythmically coordinating with a partner can increase pro-sociality, but pro-sociality does not appear to change in proportion to coordination success, or particular classes of coordination. Pro-social benefits may have more to do with simply coordinating in a social context than the details of the actual coordination (Cross et al., 2016). This begs the question, how stripped down can a coordination task be and still affect pro-sociality? Would it be sufficient simply to imagine coordinating with others? Imagining a social interaction can lead to many of the same effects as actual interaction (Crisp and Turner, 2009). We report the first experiments to explore whether imagined coordination affects pro-sociality similarly to actual coordination. Across two experiments and over 450 participants, mentally simulated coordination is shown to promote some, but not all, of the pro-social consequences of actual coordination. Imagined coordination significantly increased group cohesion and de-individuation, but did not consistently affect cooperation.

## Introduction

People coordinate their behavior with others every day, often unconsciously, through movements, gestures, and speech. These Coordinated Rhythmic Movements (CRMs) have many effects on our social cognitions, behaviors, and relationships; for instance, they can increase liking, rapport, entativity, cohesion, and even cooperation between individuals (Hove and Risen, 2009; Miles et al., 2009; Wiltermuth and Heath, 2009).

Surprisingly, the effect of CRM on pro-social behaviors is not directly driven by the details of a coordination task. CRM’s effect on pro-sociality is not contingent on strict in-phase synchrony (doing exactly the same thing at exactly the same time), or even on co-actors coordinating the same movements (Cross et al., 2016). Neither the degree of coordination (Launay et al., 2013; Kirschner and Ilari, 2014; Cross et al., 2016) nor the type of movement (fine or gross motor activities; Wiltermuth and Heath, 2009; see also Reddish et al., 2013) directly relates to the magnitude of post-coordination cooperation. The pro-social effects do not, therefore, seem to depend on the coordinated movements *per se*, but the fact that these movements create a joint social context for activity. A strong test of this is to remove the act of moving while preserving the joint social aspects. One way to achieve this is simply to have people imagine coordinating.

### Mental Simulation

Imagined scenarios are thought to retain essential spatial, temporal, perceptual, neural, and motoric properties of the events they represent (Decety et al., 1994; Jeannerod, 2001; Miles et al., 2014). Mental rehearsal has a positive effect on performance; and mentally practicing a motor skill enhances performance (Feltz and Landers, 1983, Driskell et al., 1994) across a variety of activities (Feltz and Landers, 1983, Driskell et al., 1994), including basketball, skiing, darts (Vandell et al., 1943; Clark, 1960; Suinn, 1972), and music learning (Coffman, 1990).

Mental simulation of social encounters can also elicit responses similar to the real social experiences (Dadds et al., 1997). For instance, Crisp and Turner (2009) found that simply imagining a positive interaction with an out-group member affects social cognition akin to actually having such an interaction. Similarly, Garcia et al. (2002) found that imagining being in a crowd reduces people’s helping behavior on a subsequent task, in line with how helping behavior in real life is reduced as a function of the number of bystanders (Latané and Darley, 1969).

Unsurprisingly, there appears to be neural and cognitive overlap in the operations that support mental simulation and overt behavior (Jeannerod, 2001; Fadiga and Craighero, 2004). Similar patterns of physiological activity are seen during mental simulations of certain behaviors as are seen during the actual physical execution of the behavior. For instance, Bird (1984) demonstrated that EMG measurements from the arm were similar when mentally rehashing ball throws as when actually executed. Furthermore, this overlap appears to extend more broadly beyond actions to experiences. For instance, the same auditory cortical areas are active during imagined versus actual experiences of listening to music (Halpern, 1988, Zatorre et al., 1996). Mental simulation of an event seems to have both neural and cognitive overlap with actually executing or experiencing that event.

Previous work has also showed that not only are people able to successfully perform imagined coordinated actions, but when they perform imagined joint action they take into account the same aspects of the other persons action as when actually performing such a joint action (Vesper et al., 2014). Vesper et al. (2014) suggest that individuals are therefore able to successfully integrate motor simulations of their own and another person’s actions into an imagined joint action.

Given this evidence, the effects of coordination on pro-sociality do not appear to be intimately tied to coordination success. People have been shown to be successful at mentally simulating joint action, and imagined actions and interactions are capable of fostering the same kinds of neural activity and behavioral consequences as actually doing those things. Thus, it is possible that CRM might be sufficient for to positively impact pro-sociality akin to actual CRM.

### Group Cohesion and Cooperation

Both group cohesion and cooperation have been shown to be greater following CRM (i.e., Wiltermuth and Heath, 2009). In fact, one explanation for how CRM influences variables like cooperation is through its cultivation or strengthening of group cohesion. Group cohesion can be defined as the bonds which link members of a group to each other and to the group as a whole. Group cohesion is often measured using group integrators (i.e., how close, similar, and connected one feels to the other individuals) and it is thought by some to mediate CRM’s effect on cooperation. For instance, Wiltermuth and Heath (2009) found that greater cooperation seen among groups of co-actors who had engaged in synchronized walking was partially mediated by greater group cohesion (see also, Wiltermuth, 2012). Although not all research supports this hypothesis, Cross et al. (2016) found no evidence that greater cooperation post-CRM was mediated by group cohesion.

In the experiments reported below, we explore whether mentally simulated synchronized walking fosters either greater cooperation and/or cohesion in comparison with mentally simulated solitary walking. We use a before and after measure of cohesion to test for within participant changes which might mediate any relationship between CRM and cooperation. Previous work exploring group cohesion (i.e., Wiltermuth and Heath, 2009) has shown only between group differences in cohesion post-task, as opposed to actual changes in cohesion at an individual level (i.e., greater cohesion within an individual post-CRM compared to pre-CRM). In this work, we measure actual changes in cohesion post-CRM within participants. While previous studies have measured cohesion only in terms of group integration (i.e., Wiltermuth and Heath, 2009; Cross et al., 2016), we use an expanded measure of group cohesion that encompasses both integration with and attraction to the group. Attraction to the group relates to social perceptions concerning the group, as well as motivations to stay in the group, and is considered a crucial component of the group cohesion construct (see Carron and Brawley, 2012).

### The Current Experiment and Hypothesis

The social consequences of CRM do not rely on strictly synchronous, identical, or gross muscular movements but seem to stem simply from people moving in time (coordinating) in some way in a social context. Mentally simulating actions can have similar consequences as performing those actions. This suggests that mentally simulating CRM might lead to similar social consequences as actually engaging in CRM. The purpose of the current study was to test whether imagined synchronized walking was sufficient to generate increased group cohesion and greater cooperation among co-actors as has been shown to result from actual synchronized walking.

The goals of the present Experiment are (1) to assess whether imagined movement is sufficient for coordination to affect group cohesion and/or cooperation and, if so, (2) to determine whether any changes in group cohesion mediate any effect on cooperation. We predict that participants who imagine walking in time with other people as opposed to walking alone will experience greater increases in post-task group cohesion and will be more cooperative compared to participants who imagine walking on their own. In line with Wiltermuth and Heath (2009), we predict that changes in pre- and post-task group cohesion will explain any relationship between coordination and cooperation.

## Experiment 1

### Methods

#### Participants

A total of 95 undergraduate psychology students at Leeds Beckett University volunteered to participate. Seven participants did not report age and gender; the analysis below is based on the remaining 88 participants (14 males and 74 females, *M*_age_ = 19.71 years, *SD_age_* = 1.62). All participants were naive to the aims of the study, did not know each other, and were given course credit for participation. The target sample size was 21–24 people per condition. A range was targeted rather than a specific number in recognition of the fact that groups could contain 3–4 people each. Sample size was determined in the design stage based on sample sizes used in similar studies in the literature (Wiltermuth and Heath, 2009; Miles et al., 2010, 2014; Wiltermuth, 2012). Results were not analyzed until data collection was completed and no additional data were collected after analysis. We report all measures, manipulations, and exclusions in this study. The experiment was approved by the Leeds Beckett University Psychology Ethics Review Board.

#### Design

The study employed an experimental design with two between-subjects factors: Imagined Movement (2 levels: imagining walking alone or imagining walking in-step with the rest of the group) and Task Enrichment (2 levels: instructions and video or just instructions). The Task Enrichment factor was included because the level of detail incorporated into an imagination scenario has been shown to modulate the magnitude of the proceeding effects. For example, the imagined contact effect is larger following vivid rather than basic imagination scenarios (Husnu and Crisp, 2010). Therefore, among half of the participants, we enriched the imagination task with visual information about the desired task (instructional videos).

#### Materials and Procedure

Upon entering the laboratory, the participants were seated in groups of three or four around a table so as they could not see each other’s written responses and were asked to not communicate with each other. They first completed a measure of mood and group cohesion consisting of the following questions:

(1) How happy do you feel right now? (1 = very unhappy, 5 = very happy)
(2) How close do you feel to the other participants? (Not at all – Very)
(3) How similar do you feel to the other participants? (Not at all – Very)
(4) How connected do you feel to the other participants? (Not at all – Very)
(5) How much would you like to see the other participants again? (Not at all – Very much)
(6) How much do you like the other participants? (Not at all – Very much)
(7) How attractive would you rate the other participants? (Not at all – Very)

Participants recorded their responses to Questions 2–7 by marking on a 180-mm continuum labeled with the end points of the response scale. The continuum response scale was used to make it more likely to detect any changes after the imagination manipulation and has been successfully used in a similar context by Lumsden et al. (2014).

Participants were then instructed to imagine walking along a corridor. In the IN-STEP conditions, participants were told to imagine walking in-step with the other participants along the corridor. In the ALONE conditions, participants were asked to imagine walking alone along the corridor. Half of each group were first shown an 8-s video clip of what the relevant movements would look like. The video for the IN-STEP condition showed two people (one male, one female, both in their early 1920s) walking along an empty corridor in-step. For the ALONE condition, the video showed either a male or a female walking alone down a corridor (participants in this condition only viewed one of the videos and the two versions were counterbalanced across experimental sessions). All videos were filmed on an iPhone, cropped to 8 s long, and had the audio removed. After viewing the video, participants were asked to close their eyes and imagine performing the appropriate action. The imagination phase lasted 2 min.

After the imagination task, participants were asked to complete a second copy of the mood and cohesion measure, along with one added question measuring how difficult they found the imagined movement task (5-point Likert scale, 1 = very difficult to 5 = very easy).

Finally, participants took part in a public goods game to measure cooperation (as used by Wiltermuth and Heath, 2009; Cross et al., 2016). The aim of the game was to collect as many points as possible and (in order to encourage meaningful competition without offering monetary rewards) the person who collected the most points had a chance to win a course-required textbook. For each round of the public goods game, participants had 10 tokens to allocate between two accounts: a private account and a public account. Every token in the private account was worth 5 points to the player who allocated that token, while every token in the public account was worth 3 points to each player in the group. The game had a total of five rounds. During each round, participants privately recorded how many tokens (out of a total of 10) they wished to allocate to each of the two accounts and then recorded their public account allocation on a separate slip which was folded and given to the experimenter. The experimenter then privately ‘tallied’ the total public account allocation and read it out. The actual amount reported was standardized to ensure it stayed the same throughout all sessions. For four person groups, results read out were as follows: Round 1 – 25 tokens, Round 2 – 26 tokens, Round 3 – 20 tokens, and Round 4 – 22 tokens. For three person groups they were as follows: Round 1 – 17 tokens, Round 2 – 18 tokens, Round 3 – 13 tokens, and Round 4 – 15 tokens. Standardized public account totals were used to gain a level of homogeny across different groups over participants’ perceptions and expectations of their group members’ choices.

### Results

We first examined the mood and task difficulty measures using a 2 (Imagined Movement) × 2 (Task Enrichment) ANOVA. No measures distributions deviated significantly from normality. There were no significant main effects or interactions for either mood change scores (mood after imagining – mood before imaging) or task difficulty (all *p*’s > 0.05). It was concluded that neither mood nor task difficulty contributed to the effects described below.

#### Cooperation

**FIGURE 1:**
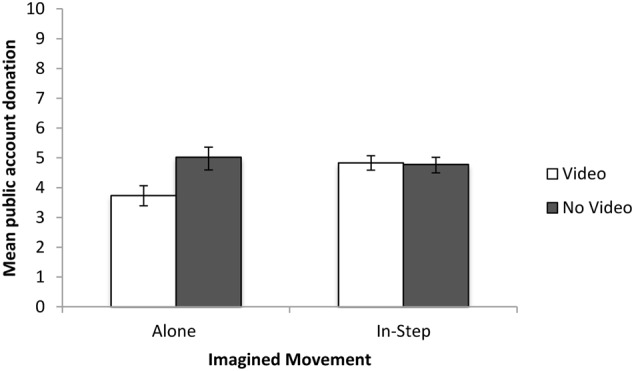
Mean and standard errors for public account donations for Experiment 1.

The data were first explored for outliers using *z*-scores in line with recommendations by Osborne and Overbay (2004). No outliers were identified, and a Kolmogorov–Smirnov (KS) test showed that none of the distributions deviated significantly from normality (all *p*’s > 0.05) and Levene’s test assumed equal variances (*p* > 0.05). We then investigated differences in cooperation (mean amount donated to the public account during the public goods game) across conditions using a 2 (Imagined Movement) × 2 (Task Enrichment) ANOVA. See **Figure 1** for the mean public account donation for each condition. There was no main effect of either Imagined Movement [*F*(1,91) = 1.67, *p* = 0.19, η^2^= 0.02] or Task Enrichment [*F*(1,91) = 3.55, *p* = 0.06, η^2^= 0.04]. However, there was a significant Imagined Movement–Task Enrichment interaction [*F*(1,91) = 4.1, *p* = 0.046, η^2^= 0.04]. The interaction showed that those who imagined walking alone after watching the instructional video cooperated *less* than those in the remaining three conditions. To confirm this, we then split the data by enrichment condition and conducted separate independent *t*-tests (with Bonferroni corrected *p*-values) comparing cooperation across imagined movement type. For the enriched condition, there was a significant difference between the cooperation scores of those who imagined walking alone and those who imagined walking in-step [*t*(44) = 2.63, *p* = 0.01, *d* = 0.77]. There was no such difference for the non-enriched conditions [*t*(47) = 0.48, *p* = 0.63, *d* = 0.14].

#### Cohesion

Recall that our measure of group cohesion included items related to both group integration and attraction to the group. To test whether all items were, indeed, related to group cohesion, an Exploratory Factor Analysis (EFA) was conducted using the pre-manipulation scores for the six cohesion questions with oblique rotation (direct oblimin). The Kaiser-Meyer-Olkin (KMO) measure verified the sampling adequacy for the analysis, KMO = 0.844. An initial analysis was run to obtain the eigenvalues for each factor in the data. Only one factor (on which all of the items loaded) had an eigenvalue over Kaiser’s criterion of 1 and it explained 62.57% of the variance. The scree plot also suggested retaining one factor as did analysis of the component matrix. See **Table 1** for factor loadings. The analysis suggests that both ‘group integration’ and ‘attraction to the group’ are indeed part of the same ‘group cohesion’ construct. On this basis, we computed cohesion composite scores (the sum of the difference, post- minus pre-manipulation, between the six cohesion change questions).

**Table 1:** The rotated loadings for the factor termed Cohesion.

	Rotated factor
	loadings
How much do you like the other participants?	**0.895**
How much would you like to see the other participants again?	**0.824**
How close do you feel to the other participants?	**0.811**
How similar do you feel to the other participants?	**0.804**
How connected do you feel to the other participants?	**0.699**
How attractive would you rate the other participants?	**0.693**

**FIGURE 2:**
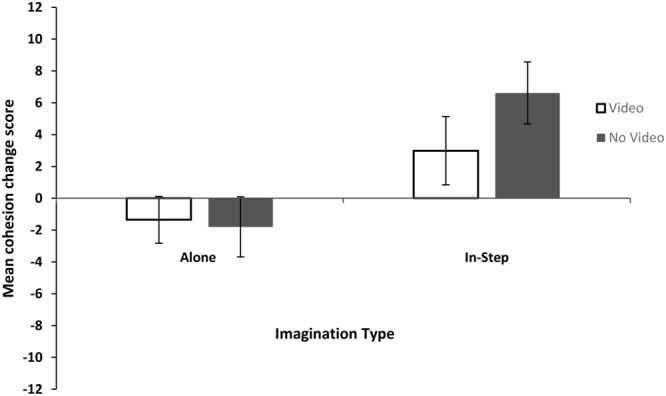
Mean and standard errors for Cohesion change scores for Experiment 1.

The data were first explored for outliers using *z*-scores in line with recommendations by Osborne and Overbay (2004). One *z-*score above the recommended cutoff point of 3 was identified (Participant 70, *z* = 3.789), and so this score was excluded from further analysis. KS tests showed that none of the distributions deviated significantly form normality (all *p*’s > 0.05), and Levene’s test assumed equal variances (*p* > 0.05). We then investigated differences in group cohesion across conditions using a 2 (Imagined Movement) × 2 (Task Enrichment) ANOVA. See **Figure 2** for the mean cohesion change scores for each condition. There was only a main effect of Imagined Movement [*F*(1,90) = 11.607, *p* = 0.001, η^2^= 0.11]; those who imagined walking in-step experienced significantly greater increases in group cohesion than those who imagined walking alone. The main effect of Task Enrichment [*F*(1,90) = 0.719, *p* = 0.39, η^2^< 0.01] and the interaction [*F*(1,90) = 1.186, *p* = 0.279, η^2^< 0.01] were not significant. Separate one sample *t*-tests confirmed that the IN-STEP condition’s mean cohesion change scores were significantly different from 0 [*t*(46) = 3.6, *p* = 0.001, *d* = 0.53] but that the ALONE condition’s were not [*t*(46) = –1.22, *p* = 0.23, *d* = –0.18].

This pattern of results differs from the pattern of cooperation scores. In the cooperation analysis, neither Imagined Movement nor Task Enrichment had an independent effect on post-task cooperation. Those in the video-enriched condition who imagined walking alone were less cooperative than those in the other conditions. In the cohesion analysis, Imagined Movement did have an independent effect on cohesion; group cohesion increased after imagining walking in-step but not after imagining walking alone, and this pattern was independent of the Task Enrichment condition. All Means, SDs, and 95% Confidence Intervals for Experiment 1 can be found in **Table 2**.

**Table 2:** Means, SDs, and 95% CIs for cohesion and cooperation scores for Experiment 1.

		Public account donation			Cohesion change score		
		Means	*SD*	95% CIs	Means	*SD*	95% CIs
Task enriched	In-Step	4.83	1.16	(4.32,5.33)	2.99	8.72	(–0.77,6.75)
	Alone	3.73	1.62	(3.03,4.43)	–1.35	7.05	(–5.11,2.41)
Non-enriched	In-Step	4.78	1.42	(4.19,5.37)	6.62	9.59	(2.94,10.30)
	Alone	5.02	2.07	(4.15,5.9)	–1.80	10.55	(–5.49,1.88)

### Discussion

Previous CRM research has restricted the operationalization of group cohesion to include only questions concerning group integrators (similarity, closeness, and connectedness). The analysis reported here suggests that group cohesion also involves the desire to see the group again and social perceptions of the group (group attractions) as suggested by Carron and Brawley (2012). Participants who imagined walking in-step with each other experienced significantly greater increases in group cohesion than those who imagined walking alone. This is not only the first study to show that imagined CRM can increase cohesion, but also to show actual increases in group cohesion post-CRM (albeit imagined CRM), as opposed to group differences. While we did observe a significant increase in cohesion post-imagined CRM, this was not accompanied by greater cooperation, scores on cohesion, and cooperation measures produced very different patterns of results. Therefore, results do not support the idea that changes in group cohesion mediate the effect of CRM on cooperation.

Without a video demonstration of the movement task, imagining walking in-step did not promote greater cooperation than imagining walking alone. With a video demonstration, imagining walking alone seemingly negatively impacted cooperation compared to all other conditions. This suggests that a suitably enriched imagined solitary movement task may be capable of decreasing cooperation/increasing competitiveness. The result is somewhat surprising given that the effect is usually framed as coordination doing something positive rather than solitary action doing something negative.

Why might imagined solitary walking decrease cooperation? Firstly, it is worth noting that cooperation was only measured post-task, so any baseline differences in cooperation between groups cannot be definitively ruled out. Although measuring cooperation using an economic game only after an experimental task is common place in the literature (Wiltermuth and Heath, 2009; Reddish et al., 2013, 2014; Cross et al., 2016).

Another potential explanation is that adequately mentally simulating walking alone (when the imagined scenario was suitably enriched) could have primed participants to think of themselves more as unique individuals. The process of an individual seeing themselves as a unique individual rather than as a member of a group is called de-individuation (Hutter et al., 2013). The combination of watching a video of solitary walking followed by imagining themselves walking alone may have led participants in this condition to think of themselves in more individualized ways, leading to more individualistic (competitive) choices in the economic game. However, this explanation was formulated *post hoc*. Results found relating to cooperation were not in line with our original hypothesis, the significance level was very close to the cutoff (0.046), and our sample size was small and a potentially limiting factor. Therefore, we do not feel meaningful conclusions can be drawn from this data alone. The relationship between imagined coordination and cooperation along with the potential role of de-individuation is further explored in Experiment 2.

## Experiment 2

The results of Experiment 1 suggest that, while imagined coordination increases group cohesion, it does not impact cooperation in the same way. Cooperation differed between the imagination conditions only when preceded by a video demonstrating to be imagined action. We hypothesized that this effect may have been due to an increase in de-individuation following mentally simulated solitary walking, though this explanation was developed *post hoc*. The purpose of Experiment 2 is to replicate the pattern of coordination/cohesion results from Experiment 1 and to explore whether de-individuation can account for these results. To enhance the generalizability of the findings, Experiment 2 was conducted in a different location, with a different population, and a much larger sample size.

Consistent with the pattern of results reported in Experiment 1, we expected that group cohesion would increase following mentally simulated CRM compared to mentally simulated solitary movement. Similarly, we predicted that participants would view themselves in less individualized ways post-CRM. We also predicted that cooperation would not be greater following mentally simulated CRM (cf. mentally simulated solitary walking), regardless of task enrichment. However, if significantly less cooperation was observed in the enriched solitary walking condition (cf. all another conditions) than this was hypothesized to be coupled with an increase in de-individuation to account for the lower cooperation.

### Methods

#### Design, Materials, and Procedure

The design, materials, videos, and procedure were identical to those used in Experiment 1 with the following exceptions:

A measure of de-individuation was added, and this measure was adapted from Hutter et al. (2013). It was scored on a 180-mm continuum and was delivered, alongside the cohesion measure, before and after the imagination task.

(Q1) How much do you see yourself as (an individual – a group member)?
(Q2) To what extent do you think of yourself as a unique individual (not at all – very much so)? (this item is reverse scored)
(Q3) To what extent do you qualify as a group member (not at all – very much so)?

Questions relating to mood and task difficulty were not included since neither variable was affected by the imagination task in Experiment 1.

#### Participants

A total of 356 students and staff members at Sunway University volunteered to participate (177 males and 179 females, *M*_age_= 19.57 years, *SD*_age_= 4.02, range 18–60 years). Sunway is an international English-speaking university based in Kuala Lumpur, whose student body is primarily comprised of Malaysian born individuals (about 90%). All participants were naive to the aims of the study. Participants were each paid MR 10 (approximately £2) for their time and the person who collected the most points had a chance to win a voucher worth MR 250 (£45). Sample size was determined in the design stage via power analysis resulting in a recommendation of 80–90 people per cell to be adequately powered to find interaction effects. Results were not analyzed until after data collection was completed and no additional data were collected after analysis. We report all measures, manipulations, and exclusions in this study. The experiment was approved by the University of Houston International Review Board and Sunway University.

### Results

#### Cooperation

The data were first analyzed for outliers using *z*-scores in line with recommendations by Osborne and Overbay (2004). No outliers were identified, and a KS test showed that none of the distributions deviated significantly form normality (all *p*’s > 0.05), Levene’s test assumed equal variances (*p* > 0.05). We then investigated differences in cooperation (mean amount donated to the public account during the public goods game) across conditions using a 2 (Imagined Movement) × 2 (Task Enrichment) ANOVA. There was no main effect of either Imagined Movement [*F*(1,352) = 1.1, *p* = 0.296, η^2^< 0.01]; or Task Enrichment [*F*(1,352) = 0.001, *p* = 0.996, η^2^< 0.01], and there was also no interaction [*F*(1,352) = 2.16, *p* = 0.142, η^2^< 0.01]. See **Table 3** for the mean public account donation for each condition. It was therefore concluded that imagined coordination had no effect on cooperation.

**Table 3:** Means, SDs, and 95% CIs for cooperation, cohesion, and de-individuation scores for Experiment 2.

		Public account donation			Cohesion change score			De-individuation change scores		
		Means	*SD*	95% CIs	Means	*SD*	95% CIs	Means	*SD*	95% CIs
Task enriched	In-Step	5.22	2.1	(4.66,5.55)	8.16	17.13	(4.49,11.83)	3.5	15.92	(0.354,6.64)
	Alone	4.86	2.25	(4.73,5.68)	–2.27	18.42	(–5.96,1.43)	1.0	11.49	(–2.18,4.18)
Non-enriched	In-Step	5.1	2.23	(4.97,5.91)	7.95	17.37	(4.26,11.65)	9.34	17.46	(6.17,12.5)
	Alone	5.44	2.19	(4.4,5.32)	–2.99	17.51	(–6.68,0.70)	1.67	14.38	(–1.49,4.83)

#### Cohesion

**FIGURE 3:**
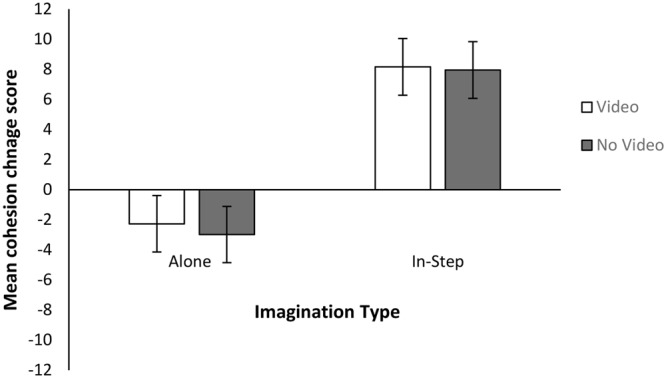
Mean and standard errors for Cohesion change scores for Experiment 2.

Cohesion composite scores were computed as in Experiment 1. The data were then analyzed for outliers using *z*-scores in line with recommendations by Osborne and Overbay (2004). Three *z*-scores above the recommended cutoff point of 3 were identified (participants 79: *z* = 3.05, 115 – *z* = –3.51, 168 – *z* =, 299 – *z* = 3.07), and these scores were excluded from further analysis. KS tests showed that none of the distributions deviated significantly form normality (all *p*’s > 0.05), and Levene’s test assumed equal variances (*p* > 0.05). We investigated differences in group cohesion across conditions using a 2 (Imagined Movement) × 2 (Task Enrichment) ANOVA. There was only a main effect of Imagined Movement [*F*(1,349) = 32.46, *p* < 0.001, η^2^= 0.09]; those who imagined walking in-step experienced greater increases in group cohesion than those who imagined walking alone. Neither the main effect of Task Enrichment [*F*(1,349) = 0.061, *p* = 0.061, η^2^< 0.01] nor the interaction [*F*(1,349) = 0.019, *p* = 0.89, η^2^< 0.01] were significant. See **Figure 3** for the mean cohesion change scores for each condition. Separate one sample *t*-tests confirmed that the IN-STEP condition’s mean cohesion change scores were significantly different from 0 [*t*(176) = 6.23, *p* = 0.001, *d* = 0.47] but that the ALONE condition’s were not [*t*(175) = –1.944, *p* = 0.053, *d* = –0.15]. All Means, SDs, and 95% Confidence Intervals for Experiment 2 can be found in **Table 3**.

#### De-individuation

De-individuation composite scores were first computed as the sum of the three de-individuation change (after manipulation – before) items (with item 2 being reverse coded). Positive de-individuation change scores indicate that participants identified more as a part of a group after the imagination task; negative de-individuation change scores indicate that participants identified more as an individual after the imagination task. The data were first analyzed for outliers using *z*-scores in line with recommendations by Osborne and Overbay (2004). Eight *z*-scores above the recommended cutoff point of 3 were identified (participants 20: *z* = 3.02, 45 – *z* = –3.35, 56 – *z* = –4.71, 94 – *z* = –3.44, 168 – *z* = –3.78, 265 – *z* = 4.89, 284 – *z* = 4.07, 299 – *z* = 3.54); these scores were excluded from further analysis. However, KS tests showed that the distributions still deviated significantly from normality (all *p*’s < 0.05). We therefore performed an Aligned Rank Transformation (ART) in line with recommendations by Wobbrock et al. (2011). This method aligns data before applying averaged ranks, making the data suitable for ANOVA [see Wobbrock et al. (2011) for a more detailed description of the ART procedure and links for tools to apply it].

**FIGURE 4:**
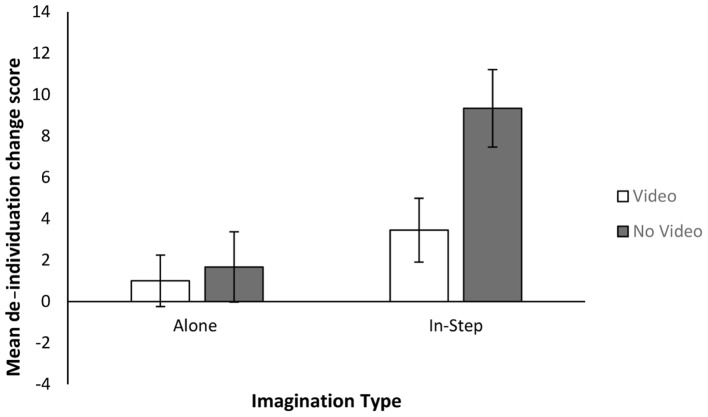
Mean and standard errors for de-individuation change scores for Experiment 2.

We then investigated differences in ART de-individuation change scores using a 2 (Imagined Movement) × 2 (Task Enrichment) ANOVA. There was a main effect of Imagined Movement [*F*(1,344) = 5.85, *p* = 0.016, η^2^= 0.02]; those who imagined walking in-step saw a greater shift in thinking of themselves as group members than did those who imagined walking alone. There no significant main effect of Task Enrichment [*F*(1,344) = 0.140, *p* = 0.709, η^2^< 0.01]; or interaction [*F*(1,344) = 1.775, *p* = 0.184, η^2^< 0.01]. See **Figure 4** for the mean de-individuation change scores for each condition. Separate one-sample Wilcoxon signed rank tests confirmed that the IN-STEP condition’s mean de-individuation change scores were significantly different from 0 (*z* = 5.259, *p* < 0.001, *r* = 0.4), but that the ALONE conditions were not (*z* = 1.875, *p* = 0.061, *r* = 0.14). Imagining walking in-step caused people to become less individuated (more group oriented).

#### Cohesion–De-individuation Relationship

Finally, we explored whether changes in cohesion scores were related to changes in de-individuation scores. We ran four separate Spearman’s correlations, one for each condition. For those who had imagined walking in-step cohesion change was positively related to de-individuation change, whether the task was enriched or not (Task-enriched – *rs* = 0.459, *p* < 0.001, Task-not-enriched – *rs* = 0.399, *p* < 0.001). For those who had imagined walking alone cohesion change was not related to de-individuation change, regardless of whether the task was enriched or not (Task-enriched – *rs* = 0.189, *p* = 0.082, Task-not-enriched – *rs* = 0.180, *p* = 0.098). Therefore, regardless of task enrichment, among those who imagined walking in sync, an increase in cohesion was accompanied by an increase in perceiving oneself as a group member rather than as an individual.

### Discussion

The results of Experiment 2 confirmed that there was no effect of mentally simulated CRM on cooperation, regardless of task enrichment. This suggests that the apparent decrease in cooperation post mentally simulated solitary walking seen in Experiment 1 is due to a type 1 error (see following section). In contrast to cooperation, group cohesion was affected by imagined movement. Those who had imagined walking in-step saw significant increases in cohesion following the imagination task (across both experiments).

Experiment 2 also explored whether imagined movement had any effect upon whether participants viewed themselves more as group members rather than individuals. Results showed that Imagining walking in-step significantly reduced de-individuation following the imagination task. That is, imagining walking in-step with others increased the extent to which people viewed themselves as part of a group, rather than as individuals. Changes in de-individuation were also found to be related to changes in group cohesion among those who had imagined walking in-step. This suggests that some of the social effects of CRM may be related to changes in how co-actors construe themselves in group/individual terms.

## General Discussion/Summary

There is now quite a lot of evidence that the effects of CRM on pro-sociality do not depend on the kinematic details of the coordination (Launay et al., 2013; Kirschner and Ilari, 2014; Cross et al., 2016). This suggests that the pro-social consequences arise from simply taking part in a joint, social activity. It is also well established that imagining social activities can lead to the same outcomes as actually taking part in the activity (i.e., Crisp and Turner, 2009). Vesper et al. (2014) showed that people can successfully mentally simulate coordinated actions; here, we show that imagining taking part in synchronized walking can lead to some of the same pro-social effects as actual synchronized walking.

Group cohesion was shown to be reliably affected by imagined CRM across both experiments. In both Experiment 1 and Experiment 2, significant increases in group cohesion followed mentally simulated CRM, whereas those who imagined walking alone experienced no change in cohesion. Although it is worth noting that the results of Experiment 2 for the ALONE condition were close to the cut-off point (0.053). This could be considered as marginally significant and is a potentially important trend as it highlights the importance of selecting appropriate control tasks when exploring the social effects of CRM^1^. Solitary versions of the coordination task are often used as control tasks in the literature (i.e., Macrae et al., 2008; Cohen et al., 2009; Hove and Risen, 2009; Sullivan and Rickers, 2013; Sullivan et al., 2014). This result suggests that comparing coordinated movements to uncoordinated group movements might make more suitable control tasks than solitary versions of the task, if we wish to be able to attribute any effects to CRM.

Aside from group cohesion, this work shows that imagined CRM leads individuals to construe themselves in more de-individualized terms. Furthermore, this increase in de-individuation was shown to be strongly correlated with increases in group cohesion. This suggests that the social effects of CRM might be related to how co-actors construe themselves in individual/interpersonal terms.

There was no conclusive evidence that engaging in mentally simulated CRM affects cooperation between people. In Experiment 1, it appeared that solitary walking lowered cooperation relative to all other conditions. However, Experiment 2 found no effects of the imagination task on cooperation. Given (1) the cooperation results in Experiment 1, which utilized a relatively small sample size, were in the opposite direction to what was predicted and (2) that this result did not replicate in the adequately powered Experiment 2, we judge the results of Experiment 2 to be the more reliable indicator of the relationship between imagined coordination and cooperation. It is likely that 1Imagined solitary walking was chosen over uncoordinated group walking here since it was suspected that mentally simulating a group of 3 or 4 people all walking with completely different gaits so as to achieve no coordination may have been too a difficult task for participants to achieve and they may have experienced a pull toward imaging synchronously walking together [since in-phase synchrony is well known to be a powerful attractor state (Kelso, 1995)]. the cooperative effects of CRM rely on a higher degree of fidelity than is afforded by imagined coordination.

## Conclusion

Mentally simulated CRM consistently increased group cohesion. Exploratory analyses suggest that this increase in group cohesion may be related to individuals starting to think of themselves in more interpersonal and less individualized ways. This work demonstrates that imagined coordination can have at least some of the same social consequences as actual coordination and these effects may be related to changes in de-individuation. Future work should now contrast mentally simulated and actual CRM in order to directly compare the social effects that are fostered by each type of task.

## Ethics Statement

Ethical approval for study 1 was granted by the Leeds Beckett University Psychology Ethics Review Board. Ethical approval for study 2 was granted by the University of Houston Institutional Review Board and Sunway University. All participants gave full informed consent by signing a consent form after reading the study information and having the chance to ask any questions.

## Author Contributions

LC collected all the data in Experiment 1 and wrote the first draft. LC and GA collected all the data in Experiment 2. LC, SG, and AW designed and analyzed Experiment 1. LC and GA designed and analyzed Experiment 2. All authors contributed to the writing of the manuscript.

## Conflict of Interest Statement

The authors declare that the research was conducted in the absence of any commercial or financial relationships that could be construed as a potential conflict of interest.
